# Supplementary figures and images for: The Urfold: Structural similarity just above the superfold level?
Source: Protein Sci. 2019 Nov 6;28(12):2119–26. doi: 10.1002/pro.3742 (PMC6863707; doi:10.1002/pro.3742)

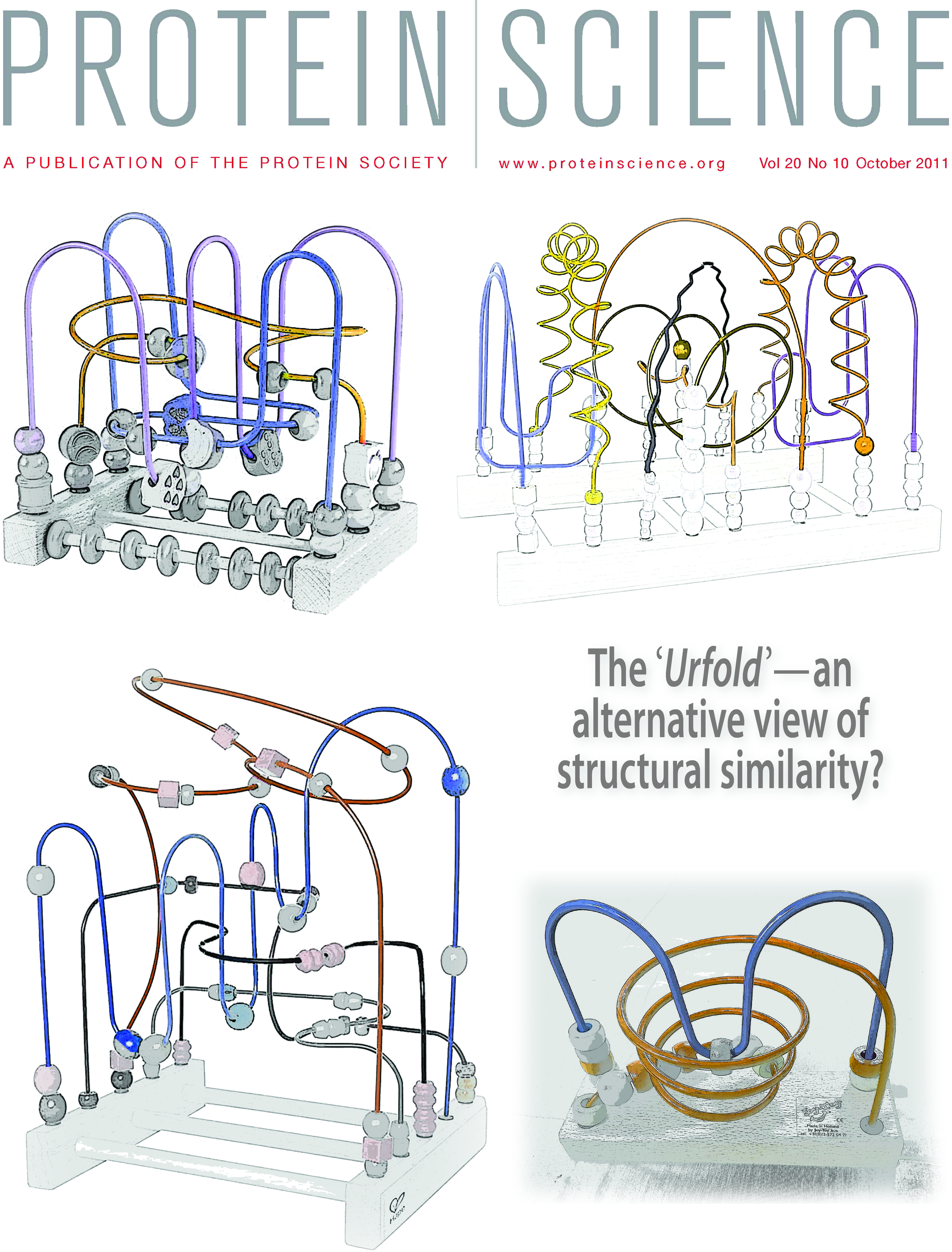

Supplement: Supplementary file 1 — Figure S1 [file PRO-28-2119-s001.tif]

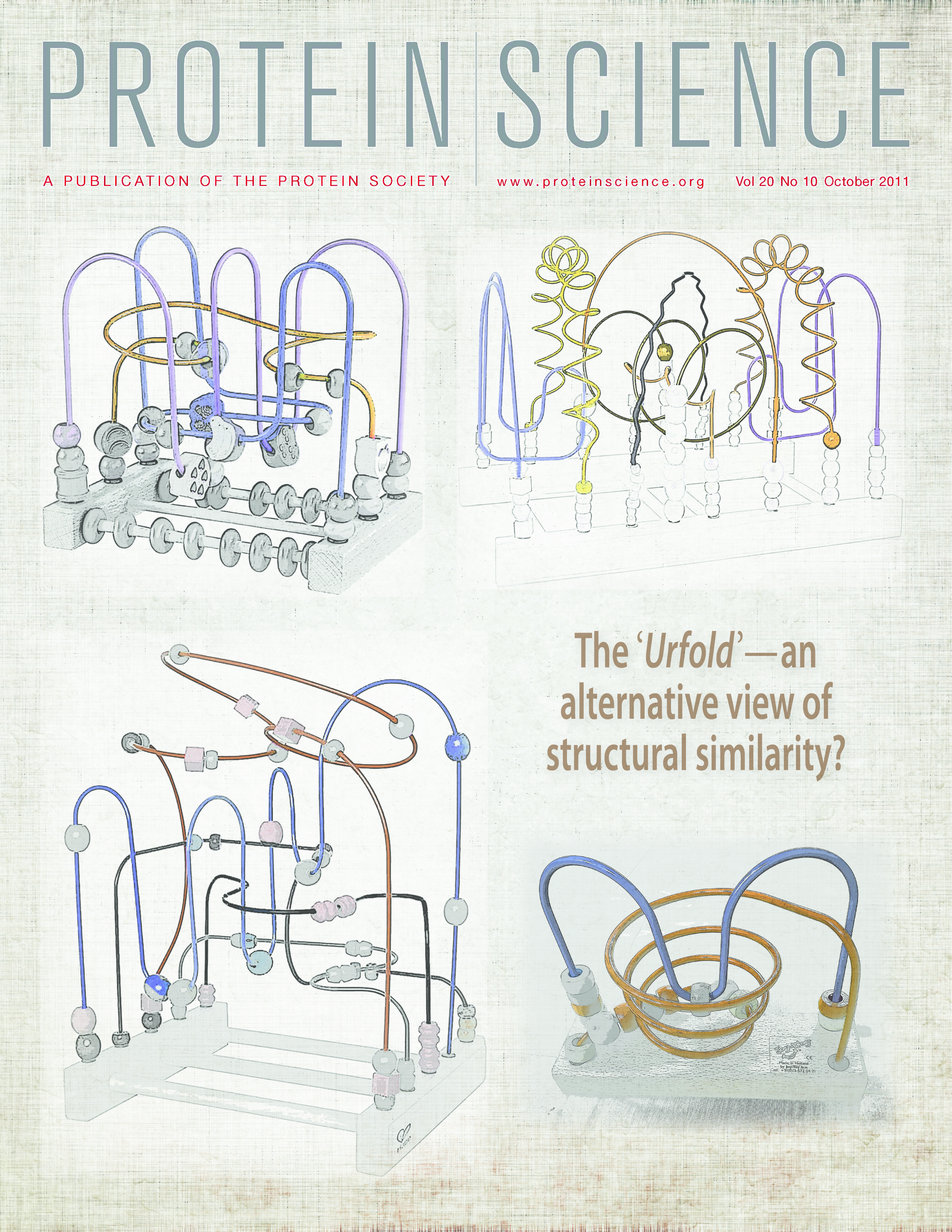

Supplement: Supplementary file 2 — Figure S2 [file PRO-28-2119-s002.tif]

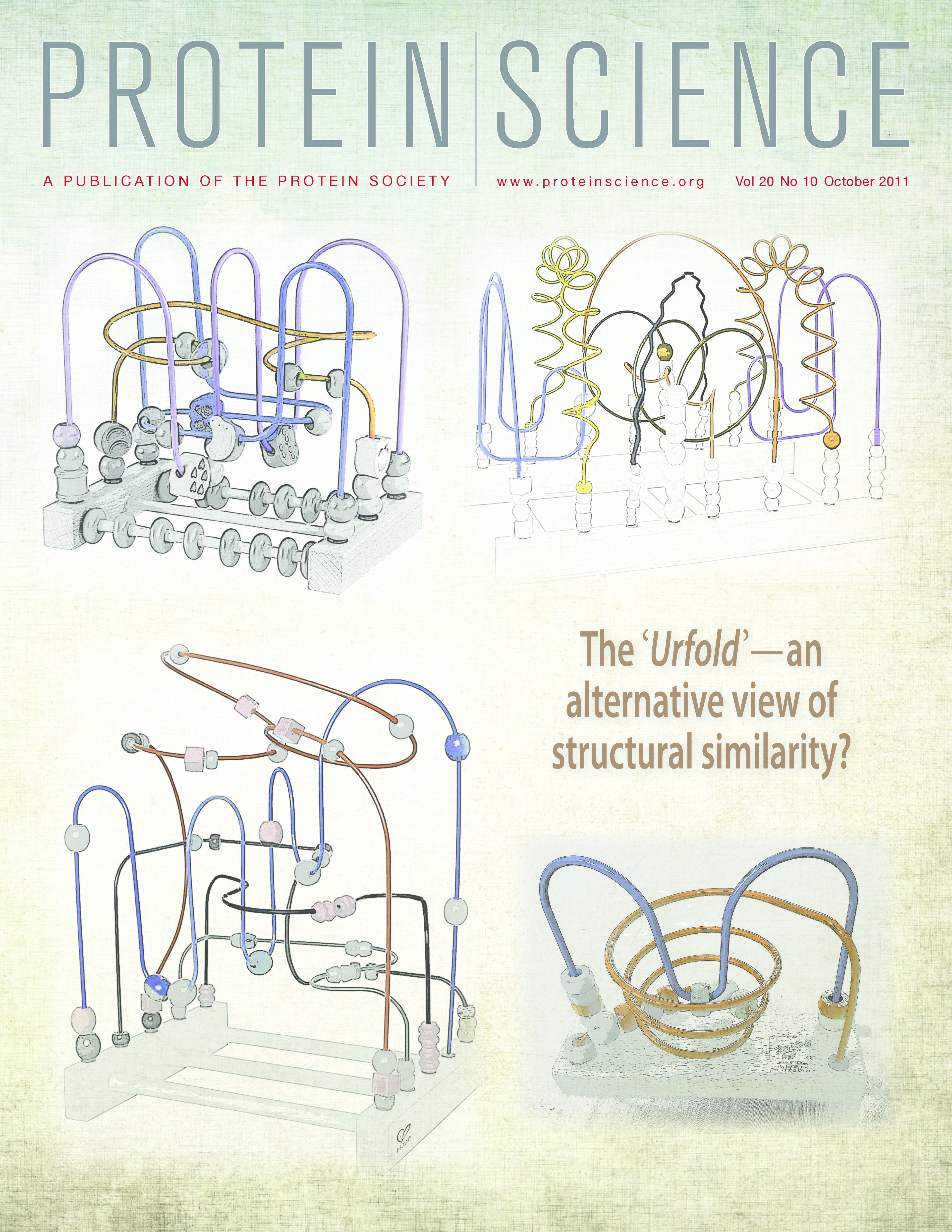

Supplement: Supplementary file 3 — Figure S3 [file PRO-28-2119-s003.tif]
